# Deep brain stimulation clinical trials: a framework to accelerate recruitment, retention, and technology translation

**DOI:** 10.3389/fnhum.2026.1851931

**Published:** 2026-07-27

**Authors:** Kara A. Johnson, Nicole R. Provenza, Sarah E. Robinson Schwartz, Jun Yu, Amanda R. Merner, Wolf-Julian Neumann, Sameer A. Sheth, Joshua K. Wong, Michael S. Okun, Megan M. Frankowski

**Affiliations:** 1 Norman Fixel Institute for Neurological Diseases, University of Florida, Gainesville, FL, United States; 2 Departments of Neurology and Neurosurgery, University of Florida, Gainesville, FL, United States; 3 Department of Biomedical Engineering, University of Utah, Salt Lake City, UT, United States; 4 Department of Neurosurgery, University of Utah, Salt Lake City, UT, United States; 5 Department of Neurosurgery, Baylor College of Medicine, Houston, TX, United States; 6 Department of Electrical and Computer Engineering, Rice University, Houston, TX, United States; 7 Department of Bioengineering, Rice University, Houston, TX, United States; 8 Neuroengineering Initiative, Rice University, Houston, TX, United States; 9 Division of Translational Research, National Institute of Neurological Disorders & Stroke, Bethesda, MD, United States; 10 Center for Bioethics, Harvard Medical School, Boston, MA, United States; 11 Department of Neurosurgery, Massachusetts General Hospital, Boston, MA, United States; 12 Wyss Center for Bio- and Neuroengineering, Geneva, Switzerland

**Keywords:** clinical trials, deep brain stimulation, neurology, psychiatry, translation

## Abstract

Clinical trials involving deep brain stimulation (DBS) are challenging to implement due to invasiveness and complexity of the therapy, substantial barriers to participant recruitment and retention, and difficulties translating emerging technologies into routine clinical practice. The 2023 Brain Research Through Advancing Innovative Neurotechnologies® (BRAIN) Workshop on Patient Recruitment in DBS Clinical Trials convened experts in clinical care, research, engineering, neuroethics, and device regulation to discuss barriers and facilitators related to recruitment, retention, and clinical translation of DBS technologies. The critical barriers to recruitment and retention included limited awareness and misinformation surrounding DBS, participant burden, and complex or inflexible trial protocols. Workshop participants identified several strategies to overcome these barriers, including improving public education, employing patient-centered study designs, engaging participants as equal stakeholders in the research, fostering partnerships with community organizations and referring clinicians, and reducing logistical, financial, and technological burdens associated with participation. Participants also identified barriers to broader clinical translation, including opaque pathways to regulatory approval and insurance coverage, access to specialized care, and adoption of new technologies. To overcome these challenges, participants emphasized the need for objective milestones, outcomes, and biomarkers, pragmatic study design, shared frameworks to support reimbursement efforts, and continued investment in multidisciplinary education and workforce development. Collectively, the workshop attendees concluded accelerating DBS innovation will require interdisciplinary collaborations among patients and patient advocates, community organizations, engineers, industry partners, researchers, clinicians, and regulatory stakeholders.

## Introduction

Deep brain stimulation (DBS) is an invasive neurosurgical procedure that involves the implantation of electrodes into specific brain regions, and electrical stimulation is delivered and controlled via an implanted battery source. The number of procedures increases each year (Johnson et al., 2024), and the FDA has approved five DBS indications since the early 2000s: Parkinson’s disease, essential tremor, epilepsy, dystonia, and obsessive-compulsive disorder (OCD). More recently, there has been a shift toward novel indications with increased research focused on non-FDA approved indications including neuropsychiatric disorders [e.g., major depressive disorder (MDD), post-traumatic stress disorder (PTSD)], non-approved movement disorders (e.g., Tourette syndrome), and cognitive disorders (e.g., mild cognitive impairment and early Alzheimer’s Disease) (Lozano et al., 2019). While recruiting and retaining participants in clinical trials is challenging (Filkowski et al., 2016; Kadam et al., 2016), DBS studies face additional hurdles given the involvement of an implanted device and the often-heterogeneous nature of the indications investigated.

This manuscript is a narrative synthesis of discussions from the 2023 Brain Research Through Advancing Innovative Neurotechnologies® (BRAIN) Workshop on Patient Recruitment in DBS Clinical Trials, held as part of the 11th Annual DBS Think Tank meeting. The hybrid workshop included experts in DBS clinical care, research, engineering, neuroethics, and device regulation and was open to public attendance. The goal of the workshop was to identify barriers and potential solutions related to participant recruitment, retention, and translation of DBS technologies into clinical practice.

### Workshop synthesis

Workshop discussions were recorded and supplemented by detailed notes taken by workshop organizers and participants. Following the workshop, a subset of authors directly involved in planning and moderating the session synthesized the discussion content into major thematic categories, including barriers and facilitators related to recruitment, retention, and translation of DBS technologies to clinical practice. Themes were identified through review of the recordings and notes and refined through discussion among coauthors. This manuscript is not intended to represent a formal qualitative analysis or consensus statement; rather, it provides a narrative summary of areas of broad agreement that emerged during the workshop while acknowledging that individual perspectives may have varied across participants. Draft sections were circulated among contributing authors for review and revision to ensure accuracy, balance, and representation of the workshop discussions.

## DBS clinical trial recruitment

**Figure 1:**
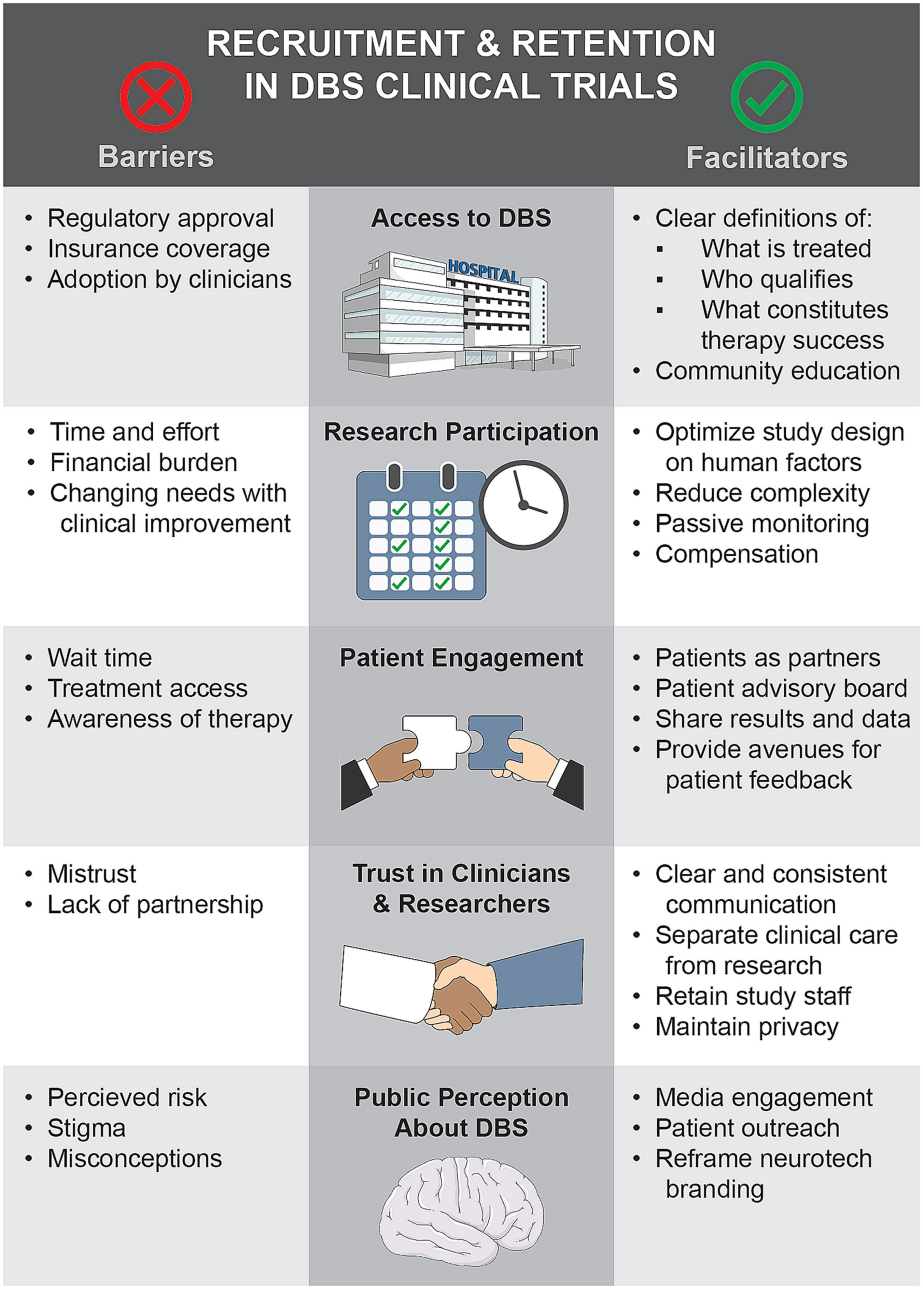
Barriers and facilitators of DBS clinical trial recruitment and retention. Summary of the key factors that play a role in participant recruitment and retention, including ensuring access to DBS, facilitating research participation, increasing patient engagement, prioritizing participants’ trust in clinicians and researchers, and improving public perception about DBS.

Recruitment is a critical yet challenging part of the clinical trial process. A 2015 analysis of 2,579 trials found that 19% of clinical trials either terminated for failed accrual or were completed with less than 85% expected enrollment (Carlisle et al., 2015). Failure to reach enrollment goals can lead to protocol and milestone delays, early trial terminations, or inability to draw conclusions at trial completion due to loss of statistical power. Similar recruitment challenges have been reported across invasive neuromodulation studies, including spinal cord stimulation trials, where investigators have identified limited eligible patient populations, restrictive enrollment criteria, participant skepticism, and substantial logistical burdens as major barriers to recruitment (Misbaah et al., 2025). These observations suggest that many recruitment obstacles encountered in DBS research may reflect broader challenges inherent to invasive neuromodulation clinical trials. The central themes and their barriers and facilitators critical for recruitment are presented in Figure 1 and discussed in details.

### Improving education for patients, clinical partners, and the community

One key barrier to patient recruitment for DBS research studies has been insufficient education or awareness about clinical trials. These gaps in knowledge include limited awareness of clinical trial opportunities, limited familiarity with the goals and significance of clinical research as well as clinical care and research investigation processes, and insufficient information surrounding what is required of trial participants in the context of implanted devices (Deuschl et al., 2006; Weaver et al., 2009; Denys et al., 2010; Goodman et al., 2010; Little et al., 2016; Holtzheimer et al., 2017; Swann et al., 2018; Vitek et al., 2020). Frequently, potential participants may resort to anecdotal accounts of their experience with DBS or recommendations from their friends, peers, acquaintances, or other sources. These n-of-1 anecdotes may sometimes yield inaccurate and unbalanced insights. Additionally, many underrepresented, socioeconomically disadvantaged, or foreign-born patients or citizens from other countries may face language barriers and other demographics-related challenges, further preventing them from being recruited for clinical trials. Indeed, studies suggest that minority groups are less likely to be asked by researchers to participate, even though they are not less likely to participate in medical research (Wendler et al., 2005).

Investigators should educate potential participants and their caregivers about their diseases, DBS, the specific technologies under investigation, and the trials’ purposes/aims. Accurate and balanced perspectives and information about DBS should be developed in collaboration with a diverse group of patient representatives and made accessible to the public, particularly given that recent research among the public has shown that, compared to other forms of neuromodulation, DBS is viewed as the most invasive, risky, and least acceptable for use (Furrer et al., 2025). Investigators should also develop strategies to educate potential community partners about DBS and effectively communicate the investigator’s efforts and intentions so that the referring physicians do not need to worry about losing their primary care patients if they decide to refer for a trial.

Additionally, investigators should facilitate transparent discussions regarding uncertain outcomes. As with many medical interventions, treatment success is defined at the group and not the individual level. This distinction is particularly important in emerging DBS indications where evidence remains limited, and patient-participants may be seeking trials in hopes of symptom relief, which is not the goal of this research stage. Care should be taken in discussions with patients to ensure they understand that participation contributes to the research needed to establish future therapies and that they play an active role in advancing the field.

#### Referral network and community engagement

A multi-pronged approach is often necessary for recruitment. Common strategies described by DBS investigators include: (1) sending letters to the patient’s providers, (2) distributing and/or displaying pamphlets and posters, (3) conducting and participating in community outreach activities, (4) advertising in institutional newsletters or local media, (5) approaching patients one-on-one in clinic, and (6) contacting patients who have indicated an interest in participating in research via patient portals (recruiting from local medical records) or listservs (recruiting from local or external databases/registries) (Filkowski et al., 2016; Vitek et al., 2020).

It is important to acknowledge that patients may prefer to hear about a DBS trial from their primary healthcare providers instead of a third party given the existing bidirectional trust in the doctor-patient relationship or with community-based organizations (Okun and Foote, 2010; Allawala et al., 2021; Shivacharan et al., 2022; Oehrn et al., 2024). Establishing a network of referring providers and reaching out to non-profits or support/advocacy groups is generally a low-cost, effective method to reach patients and improve clinical trial recruitment (Okun and Foote, 2010; Allawala et al., 2021; Shivacharan et al., 2022; Oehrn et al., 2024).

Misinformation about DBS (Little et al., 2016; Holtzheimer et al., 2017) and limited familiarity with trial eligibility criteria among referring providers may hinder recruitment and lead to inappropriate referrals (Sheth et al., 2022; Dang et al., 2023). To address the challenges and barriers, investigators should involve the medical and non-medical community partners early and throughout the trial design and conduct. Investigators should engage key community partners to participate in advisory boards or the research team itself to provide feedback on the recruitment goal and timeline, recruitment materials, marketing strategies, study design, protocols, and tools. Early and continuous collaboration may help strengthen referring clinicians’ willingness to help recruit patients (Stanslaski et al., 2012). Additionally, researchers should consider engaging a diverse group of community members, organizations, and patient advocates to provide training to research staff on cultural sensitivity and community outreach approaches.

### Positive awareness, cultural diversity, other investigational therapies, and bidirectional trust

Combating mental health stigma, patient desperation, and resistance to invasive treatments is crucial to foster bidirectional trust and acceptance of DBS as a therapy. Potential patient candidates may have a distrust and wariness of researchers, believing that the investigators are more interested in the research than in the patients’ wellbeing. Individuals may hold certain cultural beliefs and/or myths about specific diseases or therapies and may struggle with issues related to stigma. This challenge can be particularly problematic for DBS trials targeting psychiatric disorders (Tremblay-McGaw et al., 2025), where the stigma attached to mental health disorders combined with negative publicity by media and/or other sociocultural issues related to trial participation, may dissuade potential candidates from enrollment. The fear of potential complications, randomization/placebo, lack of benefit, relapse or exacerbation of symptoms, difficulty maintaining access to device-related care following the trial, and overall failure of the clinical trial can also present as obstacles to recruiting potential candidates. Additionally, other invasive investigational therapies, such as cell and gene therapies (Svendsen and Svendsen, 2024; Sartorelli et al., 2025), have made recent progress toward treating neurological disorders and could harbor interest from patients considering clinical trial participation. Other barriers include concerns regarding confidentiality, disruption of existing clinical relationships, logistical burdens, financial costs, and limited social support.

Research has suggested that establishing and maintaining honest relationships with active and former participants in DBS studies, maintaining consistent contact, and fostering bidirectional trust can help with recruitment efforts (Swann et al., 2018). Leading transparent, realistic conversations about the potential risks and benefits of participating and the trial outcomes, and in some cases how DBS may compare to other invasive investigational therapies the patient may be considering, is critical to build trust and allow participants to make an informed decision about whether to participate. Treating participants as active, willing volunteers and equal contributors to the study encourages recruitment and fosters participants’ sense of ownership in the research process (Walton et al., 2026). Constant feedback and regularly scheduled check-ins/evaluations can help study participants feel appreciated. Moreover, including caregivers in recruitment procedures and empowering them for support can be helpful. More broadly, investigators should aim to create positive awareness regarding clinical trials and should actively mitigate the misperceptions surrounding DBS.

### Develop a patient-centered recruitment plan

Developing a structured patient-centered recruitment plan prior to study initiation is fundamental for successful clinical trial recruitment. In the context of DBS, patient-centered recruitment refers to protocol development informed by patients, caregivers, and community partners to reduce burden and improve acceptability in implanted-device trials that require surgery, programming, and longitudinal follow-up. Recruitment plans should be tailored to the demographics and needs of the target population and designed to minimize participation burden whenever possible (Deuschl et al., 2006; Goodman et al., 2010). It is additionally important to establish an accurate pre-recruitment understanding of the time and resources required to recruit participants for the specific clinical trials, given the target populations, the conditions being studied, and the research protocols. Appropriate funding and personnel (e.g., a dedicated research coordinator or nurse) should be allocated to develop and execute effective recruitment strategies (Kadam et al., 2016; Lozano et al., 2019). These considerations may be particularly important in DBS studies, which often require extensive screening, frequent travel to specialized centers, caregiver involvement, and postoperative device management. One way to ensure recruitment plans are most effective and truly patient-centered is to engage with former research participants and explore motivations and deterrents to trial participation. For example, many individuals report that their primary motivation to participate is that they want to give back to science; the power of altruism and other selfless motivations can be ethically and appropriately leveraged to promote participation in DBS research as long as the participants have an adequate understanding of all potential risks involved (Dougherty et al., 2015; Luyten et al., 2016; Mosley et al., 2021; Provenza et al., 2021).

### Protocol optimization for enrollment criteria

Another commonly cited barrier to recruitment is related to study burden and protocol complexity (Deuschl et al., 2006; Goodman et al., 2010; Little et al., 2016; Holtzheimer et al., 2017; Herron et al., 2018; Swann et al., 2018). DBS trials can entail a long wait time, travel to dedicated and often remote DBS centers, extensive evaluations, and significant burdens on patient time, effort, and finances. Adopting a patient-centered approach may help address protocol-related barriers to recruitment. Enrollment criteria and protocols should be pragmatic and tailored to the specific patient population and scientific question. For example, trials focused on early-stage disease versus late-stage disease would likely require different approaches, or a trial conducted to get regulatory approval for a novel DBS indication would likely warrant a different design than a trial exploring biomarkers. Investigators should solicit feedback from former trial participants or patient advocates, caregivers, community representatives, and other key stakeholders throughout study design, including through patient advisory boards or stakeholder-engagement panels when feasible. DBS-specific outcome data on the effects of advisory board involvement on recruitment remain limited; however, broader literature suggests such engagement may improve enrollment and trial design (Crocker et al., 2018). Such approaches can help identify unnecessary participant burden, improve study acceptability, refine recruitment materials, and optimize enrollment criteria and study procedures while maintaining scientific rigor.

## DBS clinical trial retention

Clinical trials often face the competing priorities of maximizing the acquisition of clinically or scientifically meaningful data while avoiding overburdening participants. DBS trials have become increasingly sophisticated and therefore complex in design and execution over the past two decades. The landmark early trials of DBS for Parkinson’s disease, for example, used the relatively simple design of assignment to an arm receiving either DBS (in unblinded fashion) or an arm receiving best medical therapy (Deuschl et al., 2006; Weaver et al., 2009). Recent DBS trials for the same disorder, on the other hand, have adopted more complex designs incorporating implantation of all patients followed by splitting the full cohort into two or more arms in which stimulation is applied in double-blinded and sham-controlled fashion (Vitek et al., 2020). This high bar of rigorous design now seems to be a prerequisite for DBS trials, whether for approved (via U.S. FDA HDE) indications such as OCD (Denys et al., 2010; Goodman et al., 2010; Luyten et al., 2016; Mosley et al., 2021; Provenza et al., 2021) or for emerging indications such as depression (Dougherty et al., 2015; Holtzheimer et al., 2017). This increased sophistication, rigor, and complexity means greater burden on patients in terms of therapy uncertainty (whether they are on vs. off stimulation), number and type of clinical assessments, and other factors. On top of this increased clinical burden, current DBS trials have also added scientific burden. The longitudinal and complex nature of these studies incur a high financial and time cost that can lead to participation fatigue and low compliance with study protocols. These challenges may be further exacerbated by a lack of familial or social support, mistrust between the participant and study staff, unclear expectations for participation, and changing clinical status of the patient. To retain patients in these trials, it is critical to design protocols in a way that reduces burden (i.e., time, effort, and cost) and the perceived complexity of research activities. The central themes and their barriers and facilitators critical for retention are presented in Figure 1 and discussed in detail.

### Reducing participant burden

#### Time and effort burden

Current DBS trials are relying less on empiricism to demonstrate efficacy and instead are placing increasing importance on understanding mechanisms as a path to improve outcomes. This trend is being driven by both the desire to continue improving outcomes and the availability of new technologies. The field is moving toward strategies that inform therapy delivery based on neurobehavioral data (including adaptive and responsive DBS) (Little et al., 2016; Swann et al., 2018; Provenza et al., 2019; Opri et al., 2020; Gilron et al., 2021; Scangos et al., 2021; Shivacharan et al., 2022; Oehrn et al., 2024), and personalized approaches for tailoring therapy to individual needs (Okun and Foote, 2010; Allawala et al., 2021; Sheth et al., 2022; Dang et al., 2023).

The recent introduction of recording-capable DBS devices provides a technologically advanced toolkit for researchers to study intracranial activity and behavior both in the clinic during study visits and in the real world (Stanslaski et al., 2012; Herron et al., 2018; Thenaisie et al., 2021). These devices enable detection of neural biomarkers that could be used to inform therapeutic decision-making or enable adaptive DBS systems to automatically adjust stimulation parameters based on symptom state. However, to identify biomarkers, it is critical to collect large datasets of neural activity that are time synchronized with behavior. These large, precious datasets are acquired during long study visits with extensive testing, requiring a great amount of time and effort from both the participants and the study team over a long period of time. Even complex protocols should be implemented in a way that is simple for study participants to understand and follow. When possible, study visits should be paired and co-located with standard clinical visits to maximize the productivity of each visit.

#### Technology burden: behavioral data collection

In the DBS field, there is an increasing interest in using ecological momentary assessments to evaluate symptoms or clinical status more frequently than during clinical visits. However, self-report ratings are high burden (and therefore temporally sparse) and suffer from subjectivity (Haeffel and Howard, 2010). For these reasons, many investigators are incorporating passive behavioral data collection via wearables into study protocols. Behavioral data collection via wearables is arguably the least burdensome for patients but may impinge on patient privacy. While there has been progress with linking these continuous, passive, data streams with clinical states, this method is still largely exploratory and more work needs to be done to validate these measures.

#### Technology burden: neural data collection

Likewise, there are various DBS devices available (and several in development) that enable onboard collection of neural data, including the Medtronic Percept (PC and RC) and Neuropace Responsive Neurostimulation (RNS). The Medtronic Percept enables time-domain streaming of neural data with a clinician-facing programming tablet as well as lower-resolution recordings saved on-device that do not require participant input or engagement (Thenaisie et al., 2021). Passive neural recording capabilities demonstrate an advantage over active recording paradigms given the reduced time and effort burden for both participants and researchers. Previous studies relying on participant engagement to conduct frequent neural streaming at home yielded variable compliance rates across studies (Kremen et al., 2018; Gilron et al., 2021; Provenza et al., 2021; Alagapan et al., 2023; Shirvalkar et al., 2023; Oehrn et al., 2024; Merk et al., 2025).

As more recording-capable DBS devices become available, protocols should be designed in a way that capitalizes on low-burden research activities and prioritizes/limits high-burden data collection based on primary research goals. Researchers could conduct remote and passive monitoring when possible, rather than requiring the patient to be actively engaged in data collection. Ideally, research activities, including both neural and behavioral data collection, would be continuously ongoing in the background of everyday life activities (Cagle et al., 2024; Fan et al., 2024; Provenza et al., 2024). This approach would both reduce participant burden and extend the ethological relevance of the data we collect.

#### Financial burden

The financial burden for study participation is often significant. Trials typically only include a limited number of sites equipped to offer DBS therapy, so participants may need to travel some distance to be able to participate. Additionally, participants may be so disabled that it is not feasible to travel without a caregiver, which essentially doubles the cost of travel. Compounding the situation, participants and their caregivers may have to take days off from work or unpaid leave to attend study visits. These costs likely impact participants’ abilities to maintain compliance with study protocols throughout the long study duration. To offset these costs, researchers could build in a financial reimbursement structure into the study protocol that covers the cost of travel and time for required study visits. There could also be a financial incentive structure for completing any research activities that are required outside of the clinic (e.g., a small amount of compensation for each day a wearable device is worn).

#### Decreased patient compliance due to clinical factors

In some cases, the clinical status of the participant may interfere with their ability to complete research activities. For example, when symptoms are more severe, participants may be physically, emotionally, or cognitively unable to complete research-related tasks or activities. At the fortunate other end of the spectrum, patients who have responded significantly to DBS may not feel the need to return to see the investigator team on a regular basis and may lose motivation to continue participating in the study protocol. This phenomenon may be more common in younger populations who have the greatest number of competing priorities as their symptoms improve, including reengaging in higher education, professional careers, and social relationships. Researchers investigating DBS could consider building flexibility into research protocols to provide adequate support for completion of research activities during symptom exacerbations and accommodate changing participant priorities following clinical improvement. When scientifically and regulatorily appropriate, strategies such as remote assessments, telemedicine visits, flexible scheduling, caregiver involvement, and alternative methods of data collection may help reduce participant burden while preserving study integrity.

#### Long-term device management

Another consideration unique to DBS clinical trials is the long-term management of implanted devices after trial completion or termination. Unlike most pharmacologic interventions, DBS trials may leave participants with an implanted system regardless of whether the investigational therapy ultimately demonstrates efficacy or receives regulatory approval. Barriers to facilitating post-trial device management have been cited as including limited funding, insufficient specialized clinical infrastructure, and challenges in maintaining relationships with stakeholders (Higgins et al., 2024). Investigators should establish and communicate a clear post-trial management plan before enrollment, including continued clinical follow-up, device programming, battery replacement, potential explanation, financial responsibility for ongoing care, and circumstances under which stimulation may be continued or discontinued (Sankary et al., 2022; Okun et al., 2024). These discussions should be incorporated into the informed consent process and revisited throughout the study to establish realistic expectations regarding post-trial care. Intentional planning for long-term device management not only supports ethical conduct of DBS research but may also strengthen participant trust, improve retention, and facilitate responsible translation of investigational therapies into clinical practice.

## Fostering trust

Long-term DBS studies involve repeated visits for device programming and measuring biomarkers and outcomes over a long period of time, which inevitably produce years-long relationships between patients and the study team throughout a stressful time in the participants’ lives. Trust should be established early on and be maintained throughout the course of the study protocol and treatment. Ideally, participants should feel that they are equal stakeholders in the research to maintain a sense of accomplishment and positive outlook throughout the study.

### Clear and consistent communication

Clear, supportive, and consistent lines of communication between patients and study staff are essential for retention. This includes setting reasonable expectations in terms of clinical response to DBS, hopes for the trial, time commitments, and compliance. The design of DBS studies often includes a period when patients are blinded to stimulation condition or undergo a blinded discontinuation of stimulation therapy. These periods contribute to fears of adverse events and side effects and if implemented poorly may create mistrust between the participant and the study team.

While a large team of study staff may be required to maintain longitudinal, high-density visit schedules for many participants, the most consistent forms of communication are through a single or small number of representatives from the study team. Retaining study staff is essential for maintaining consistent communications with participants. A lack of retention of clinical coordinators may lead to inconsistencies over time that may further exacerbate mistrust and compliance and retention issues in DBS studies. Further, it is important to communicate and reinforce that clinical care is in no way dependent on compliance with research activities. Patients may feel like research activities are prioritized over their clinical care, which calls for a clear delineation between clinical and research teams.

### Participants as equal stakeholders in research

Creating an environment in which participants feel like they are active partners in the research is essential for establishing trust. In DBS studies, this may include soliciting feedback regularly from participants regarding surgical experiences, device programming, longitudinal data collection procedures, and overall study burden, sharing study findings with participants, and creating a patient advisory board for protocol design (Pham et al., 2026).

### Improving public perception of DBS

Stigma, including public perception of DBS and negative or misinformed media coverage, can impact participants’ feelings about study participation and trust with the study team (Merner and Kubu, 2023). The relationship between the study team and the participant can be harmed by bad press, even from other groups (in the same field). Conversely, informed media coverage can instill trust between participants and the study team. Researchers would benefit from engaging with the media to cover DBS trial outcomes, publications, and technological innovations that result from the study. Not only is this press good for career advancement of the research team, but it may be encouraging for participants. As researchers, it is our ethical responsibility to engage with and help guide the narrative surrounding DBS therapy.

## Translating DBS research to the clinic

**Figure 2:**
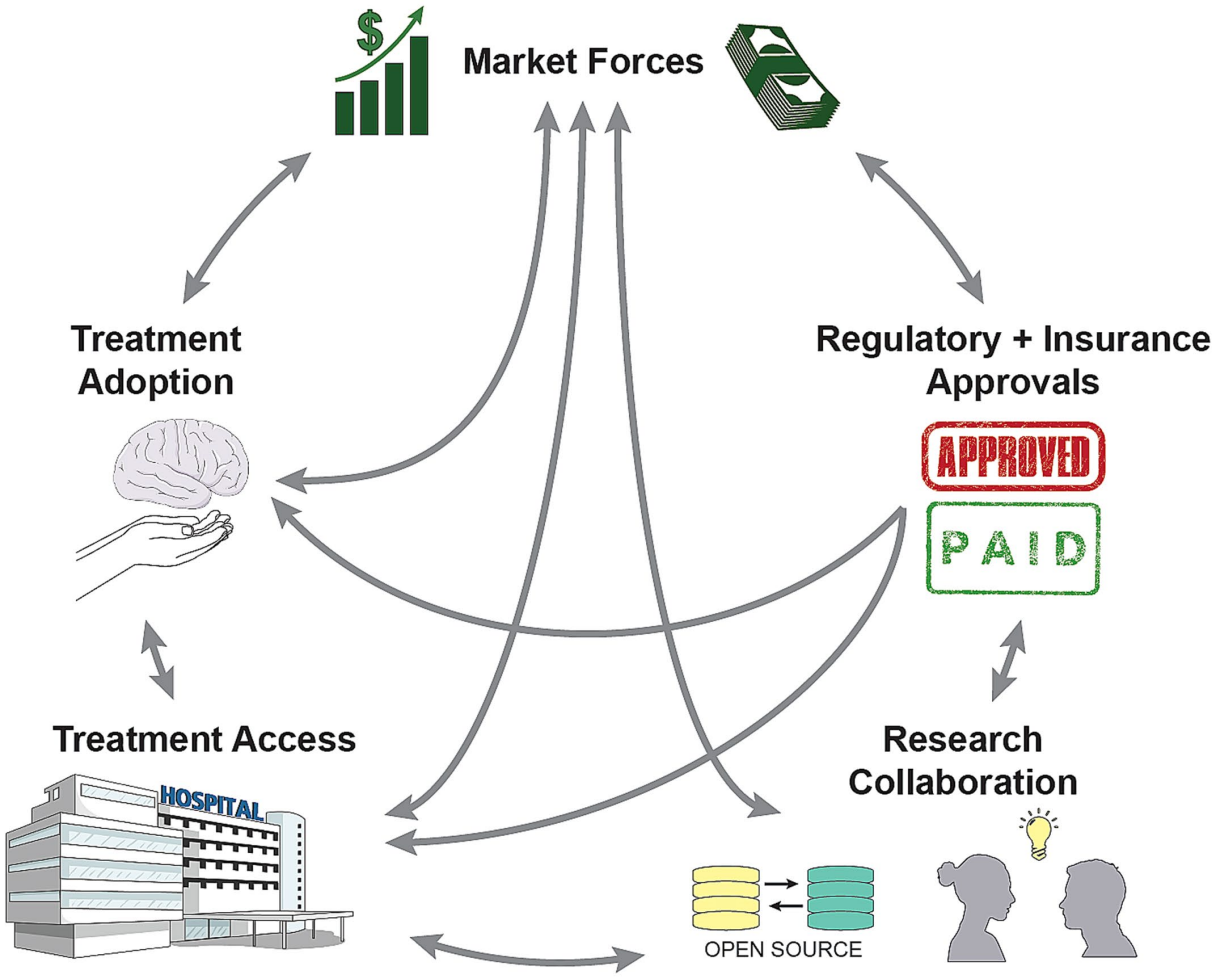
Interdependencies across factors affecting DBS technology translation. Market forces, regulatory and insurance institutions, multidisciplinary research collaboration, access to DBS, and adoption of DBS by the public and clinicians are critical and interrelated factors that impact the translation of novel technology to clinical use.

Translating new approaches and technological advancements to the clinic is a crucial step in establishing DBS as a widespread therapy for neurological and psychiatric disorders. However, translation is a challenging and intensive process that is driven by several complex and interdependent factors. The main barriers are described below, and their interdependencies are depicted in Figure 2.

### Regulatory approval and insurance coverage

One major barrier in translating new therapies to the clinic is obtaining regulatory approval for clinical use, which requires investing substantial time, money, and effort toward preclinical and clinical studies to prove safety and efficacy. As the complexity of devices has increased over time, so has the complexity of medical device regulation by the US Food and Drug Administration (FDA) (Darrow et al., 2021). Furthermore, approval processes and policies differ across regulatory agencies. Obtaining regulatory approval for clinical use is necessary for the therapy or device to be covered by private medical insurance, Medicare, or Medicaid. Approval and insurance coverage of DBS are crucial for enabling patient access to the therapy, increasing patient and clinician adoption of the therapy, and supporting widespread research collaboration, which all ultimately influence market forces. Finally, some DBS indications such as Tourette syndrome have not been granted approval by regulatory authorities due to the relatively small eligible patient population.

Unfortunately, regulatory approval does not always guarantee widespread clinical adoption. The experience of anterior nucleus of the thalamus (ANT) DBS for drug-resistant epilepsy illustrates this challenge. Following the SANTE trial, ANT DBS received FDA approval and has demonstrated sustained efficacy and safety in both long-term follow-up studies and real-world registries (Peltola et al., 2023; Remore et al., 2023). However, successful implementation of ANT DBS requires specialized multidisciplinary expertise in patient selection, surgical targeting, device programming, and longitudinal epilepsy management, which has limited availability across centers. Additionally, patients and clinicians must navigate an increasingly complex neuromodulation landscape that includes vagus nerve stimulation and RNS. These observations highlight the importance of considering implementation and dissemination strategies throughout the translational pathway rather than viewing regulatory approval as the endpoint of clinical development.

Even when a therapy is adopted clinically, regulatory approval does not necessarily translate into insurance coverage and patient access. DBS for obsessive-compulsive disorder (OCD) received FDA approval in 2009 under a humanitarian device exemption (HDE), which is reserved for therapies applicable to very limited patient populations. Despite its regulatory approval, patient access to DBS for OCD has been severely limited (Davis et al., 2021; Visser-Vandewalle et al., 2022). A recent study reported fewer than 50% of patients who qualified for DBS for OCD received the therapy, with lack of private insurance coverage as the most common limiting factor (Pinckard-Dover et al., 2021). In a separate recently published cohort of 21 patients, nine of 21 (43%) were initially denied coverage (Bentley et al., 2022). Together with the experience of ANT DBS for epilepsy, these findings highlight that regulatory approval alone is insufficient to ensure successful translation and that implementation, reimbursement, and patient access remain critical determinants of clinical adoption.

#### Develop indication-specific strategies and shared frameworks

To aid in the regulatory approval process it is crucial to be strategic about finding paths of “least resistance” to efficiently gather the required safety and efficacy data. Additionally, it is important to establish clear definitions of what specifically is being treated with DBS (e.g., specific symptom or disorder), who qualifies, and what constitutes therapy success. Facilitators to aid in obtaining insurance coverage include reducing stigma of surgical therapies (especially for psychiatric indications) and developing shared frameworks to petition for insurance coverage (e.g., discussing strategies and sharing petition letter templates).

### Measuring outcomes

There is a critical need to develop techniques and markers that can be objectively measured and that are sensitive and specific to the symptoms or disease being measured. However, identifying objective symptom biomarkers to guide clinical decision-making requires extensive investigation and validation. If a new biomarker is to be used to guide DBS therapy, it must be sensitive to changes in disease state over time, demonstrate adequate sensitivity/specificity, and be directly compared against control conditions and/or populations. Potential biomarkers may include neurophysiological signals, wearable sensor-derived measures of behavior, or other digital biomarkers that quantify symptoms. Control conditions may include on/off medication or on/off DBS, and control populations depend on the specific scientific question. Importantly, new biomarkers must ultimately be validated against established “gold standard” outcome measures with sufficient accuracy to test whether they reflect established clinical evaluations of diagnosis and symptom severity.

While challenging to develop, objective biomarkers could transform care by providing standardized, data-driven measures that minimize bias associated with patient-reported or clinician-scored outcomes. These data-driven approaches could reduce the burden on clinicians by eliminating or supplementing clinical rating scale administration and identifying therapeutic DBS paradigms more quickly, thereby reducing the burden on patients by decreasing their time in clinic or self-reported data required for therapy optimization. Finally, adoption of better markers of symptoms and disease states could tie directly into obtaining regulatory approvals for clinical use by enabling us to define patient candidates based on objective criteria and define treatment success using objective, sensitive, and specific markers.

### Patient and clinician access and adoption

Recent estimates suggest that of all movement disorders who qualify for DBS, approximately 1–4% of Parkinson’s disease or dystonia patients ultimately receive the therapy (Kestenbaum et al., 2015). Access to DBS is driven by a complex combination of factors, including regulatory approval and insurance coverage, sex/race/socioeconomics, regional access to specialty clinical centers offering DBS, fear of surgery and potential adverse events, and adoption of the therapy by patients and their clinicians (Sarica et al., 2023).

#### Increasing awareness and access

Ensuring reliable insurance and Medicaid/Medicare coverage (which is tied directly to obtaining regulatory approval) is crucial for both patients and clinicians to adopt DBS as a widespread therapy. Additionally, increasing awareness about DBS through advertising and outreach in the community is important to reach a broader audience. We even discussed “rebranding” DBS using a more familiar, accessible term (e.g., “brain pacemaker”). Ultimately the goal is to simultaneously improve access to information and reduce misinformation about DBS to enhance awareness and reduce stigma in the community. Additionally, engaging patients in the conversation surrounding their needs and expectations for DBS therapy is critical. Patients may be more likely to adopt DBS if they can choose between different device features and technologies based on their functional goals or preferences (e.g., opting for rechargeable vs. non-rechargeable devices, telemedicine for remote programming).

Furthermore, DBS is currently offered at select specialty medical centers, which limits access for both patients and clinicians. Simplifying DBS through streamlined surgical workflows, more efficient postoperative programming approaches (including algorithm-assisted and remote programming), and shared-care models between specialty DBS centers and community clinics could reduce burden and increase adoption. Additionally, there could be greater investment in clinician training to optimize patient management and to reduce time/complexity of DBS. We must be able to simplify these complex research-grade approaches to fit into a daily clinical workflow for clinicians to be able to deliver DBS both effectively and efficiently. Optimizing DBS therapy delivery is also directly linked to market forces, as increasing efficiency would increase profitability, and research collaboration through making DBS a more widespread therapy across institutions.

### Market forces and stakeholder engagement

#### Investing in advertising and technology development

Increasing industry investment in marketing and outreach may be one means of facilitating increased access to DBS for a greater number of patients. Advertising in the media as well as outreach at patient support groups or foundation events can help increase awareness and reduce stigma surrounding brain stimulation therapies (potentially alongside DBS rebranding) to increase patient adoption. Another potential facilitator is to increase investments in research, technology development, and academia-industry partnerships. Expanding research to improve current DBS therapy and to expand DBS to treat new indications will be critical. Additionally, increasing collaborations among academia-industry partners would undoubtedly lead to better technology and aid in the translation process. One example of this is the National Institutes of Health (NIH) BRAIN Initiative’s Public-Private Partnership program, which aims to facilitate partnerships between clinical investigators and manufacturers of stimulation/recording devices to reduce barriers to translation. Finally, meaningfully engaging with patient stakeholders to get feedback at every stage of technology development will help ensure the needs and priorities of each clinical population are considered and integrated into device development. Prioritizing patients’ perspectives in device development can facilitate alignment between patient needs and device design and ultimately increase adoption of the technology.

### Role of academic structure

While challenges related to collaboration and data sharing are not unique to DBS, they may be particularly important in neuromodulation research, which requires coordination among clinicians, engineers, neuroscientists, industry partners, and regulatory stakeholders. Limited multicenter data sharing, fragmented datasets, non-standardized outcome measures, and proprietary software and hardware ecosystems may impede biomarker validation, replication of findings, and translation of novel DBS technologies into clinical practice.

#### Prioritize interdisciplinary collaboration and support for next-generation researchers

Facilitators to bolster collaborative research in the DBS field include promoting multicenter data-sharing initiatives, harmonizing outcome measures across studies, and encouraging development of interoperable and open-source hardware and software solutions. Additional strategies may include funding mechanisms that incentivize collaborative teams and academia-industry partnerships. Continued progress in DBS will also depend on cultivating and retaining a diverse and talented next generation of clinicians, scientists, and engineers through mentorship and career advancement opportunities.

## Conclusion

Experts in DBS clinical care, research, device regulation, engineering, and neuroethics came together during the 2023 BRAIN Workshop on Patient Recruitment in DBS Clinical Trials to discuss barriers and facilitators related to recruitment and retention in DBS clinical trials and translation of DBS research. Workshop participants identified awareness, trust, participant burden, and protocol design as major determinants of recruitment and retention. Addressing these barriers will require patient-centered study design, reduced participant burden, meaningful stakeholder engagement, and broader public education regarding DBS. Beyond recruitment and retention, widespread clinical adoption of DBS will depend on overcoming challenges related to objective outcome measurement, regulatory and reimbursement pathways, access to care, and investment in the future workforce. Progress will require coordinated efforts among patients, advocates, clinicians, researchers, industry partners, payors, and regulatory agencies. Continued interdisciplinary collaboration will be essential to accelerate DBS innovation and expand access to safe and effective neuromodulation therapies.

## Data Availability

The original contributions presented in the study are included in the article/supplementary material, further inquiries can be directed to the corresponding author.
